# Rush Medical College — Alumni Meeting and Commencement Exercises

**Published:** 1871-03

**Authors:** 


### *Rush Medical College—Alumni Meeting and Commencement Exercises.*

February 1st, 1871, was a gala day at "Old Rush." The alumni meeting was well attended, and the commencement exercises were of unusual interest.

The alumni assembled at 10 A. M., in the lower lecture room of the College, ABNER HARD, M.D., of Aurora, the President, in the chair. Over two hundred alumni reported. After the usual formal business, the association proceeded to an election of officers, with the following result:

*President*—DR. CHRISTOPHER GOODBRAKE, of Clinton, Ill.

*Vice Presidents*—DR. J. P. WALKER, of Mason City; Dr. F. A. EMMONS, of Chicago.

*Secretary*—DR. S. COLE, of Chicago.

*Treasurer*—DR. W. C. HUNT, of Chicago.

*Executive Committee*—DRS. D. S. ROOT, J. W. TOPE, R. L. LEONARD.

After some further general business, of which the Secretary has not given us the details, the officers elect assumed their positions.

Dr. Goodbrake said the honor was unexpected. He had never before met with the association, but was well acquainted with many of the graduates, and he had always found them perfect gentlemen. In three years' service during the war, he had met many of them, and always found them to be faithful in duty and skillful in their profession. The doctor concluded by again thanking the association for the honor which had been conferred upon him. The language of Shakespeare, "Some men are born great, some acquire greatness, and some have greatness thrust upon them," could be applied to himself, as far as its concluding words were concerned.

Dr. Abner Hard, of Aurora, the retiring President of the Alumni, then delivered the annual address.

After treating at some length upon the importance and responsibility of the profession, and its achievements in alleviating the distresses of mankind, the doctor concluded his very able address as follows:

Twenty years ago, as we listened to the lectures of Professors Brainard, Herrick, Blaney, Freer, and their colleagues, little did we imagine what was to be the burden of labors. The most to which we aspired was to become successful practitioners of medicine. The future was full of hope, and the teachings of our Alma Mater were not such as to discourage us. But how has it been? Not only have we been called upon to battle with disease in its complex and multifarious forms, which too often has baffled our skill and sunk us almost to the depths of despondency, but cares and responsibilities have surrounded us, which were unlooked for and unthought of. An army of quacks and pretenders, of all grades and names, have swarmed about us, and a credulous people, not only willing, but apparently anxious, to be humbugged, have been, in too many instances, ready to put their faith and their lives in these men's keeping, generally paying a high penalty therefor, and yet ready to try it again.

The press, both secular and religious, for the sake of the paltry price paid for their advertising, have lauded, and puffed, and recommended these knaves and their nostrums, to the not unfrequent fatal injury of the individual, and the demoralizing of the community, and by some strange, mysterious and paradoxical arrangement the whole responsibility of the misfortune is most commonly thrown back on to the shoulders of the regular profession. The public should be more thoroughly informed in regard to the true claims and the true position of regular medicine. Much of the mystery that hangs about it should be swept away, and the science of it should in a far greater degree be reduced to the comprehension of the common mind. In a word, might not much of it be let down from its cold scientific heights and be made popular? The public might be shown, for instance, that all the known remedial agents of any *positive* value, and all the surgical appliances of any real merit, have originated with, or are traceable either directly or indirectly to, the regular school of medicine; that the substantial scientific basis on which it rests its claims to public support and consideration have been developed and built up during the present and past ages, by the members of its own school. It might easily be shown that all the great branches of medical science, such as anatomy, physiology, surgery, pathology, and many others, the slow growth of more than twenty centuries, have been brought to their present state of brilliant perfection by the increasing toil and unremitting labors of the regular profession. And with equal ease it might be shown that all other schools are fragmentary and partial, and that the little that belongs peculiarly to any of them that is valuable, belongs equally to us.

Besides the means already indicated, each regular physician can so deport himself as to educate and elevate at least a portion of his neighbors by a well ordered life and a faithful and skillful discharge of his duties. Legislation, in my opinion, can afford us no relief, and enlightened public sentiment must, and in due time will, I am confident, do the work. "Blood will tell," is an old saying, and so also, I add, will science, so will skill, and above all, so will brains; and I think I hazard nothing in saying that while the regular schools of medicine, with all their vast, brilliant, scientific, and other achievements over the various forms of disease, while through their agency and instrumentality the average of human life has been lengthened from ten to fifteen years, and while also they are by no means, at the present time, masters of the situation, death and disease to-day baffling them on every hand.

I congratulate you, gentlemen, upon being honored members of the only true and catholic school of medicine known among men. I congratulate you upon being honored members of the most useful and scientific profession among men. I salute you as the honored sons of an illustrious and cherishing mother.

I salute you, devout and humble children, in the act of paying your annual tribute of respect and devotion at the shrine of your Alma Mater. May the sphere of her usefulness never be more circumscribed than now, but in the future, as in the past, may the star of her glory shine brighter and brighter until the perfect day; and may the time never come when her sons shall cease to hail her, salute her and love her as their cherishing mother.

At the close of the address of Dr. Hard, the alumni adjourned to the lecture room above, for the purpose of witnessing some experiments in the science of vivisection, to be made under the supervision of Prof. J. W. Freer.

[A general account of these experiments which were characterized by the usual skill, accuracy and success exhibited by Prof. Freer, will be given elsewhere by Dr. Wadsworth, his accomplished assistant.]

The evening exercises were commenced with prayer by Rev. L. C. Chamberlain.

The roll of the graduating class was then called, the President delivering them their diplomas. Their names are as follows:

E. V. Anderson, Illinois; W. W. Baxter, Illinois; W. E. Blackman, Iowa; H. S. Bachman, Michigan; T. H. Bragg, Iowa; A. L. Buchan, Wis.; G. W. Brandon, Illinois; J. M. Bartholow, Illinois; E. W. Clark, Iowa; E. J. Chapman, Indiana; F. C. Conan, Wisconsin; N. S. Craig, Iowa; C. W. Cornell, Iowa; D. B. Collins, Wisconsin; B. D. Copp, Wisconsin; J. Y. Galer, Iowa; R. C. Grigg Illinois; S. A. Greenwell, Nebraska; G. G. Goll, Illinois: W. R. Geiger, Iowa; Thomas Gilluly, Wisconsin; S. T.

Hurst, Illinois: Wm. L. Harcourt, Illinois; J. N. Hannaford, Indiana; John L. Hays, Illinois; B. R. Hall, Wisconsin; J. L. Hagerty, Indiana; C A Hudson, Tennessee; J. H. Hutchins, Wisconsin; J. V. Harris, Illinois; W. T. Montgomery, Illinois; E. N. McGary, Minnesota; J. N. Miller, Missouri; D. C. Nicosin, Illinois; George E. Newell, Wisconsin; A. T. Peck, Wisconsin; John F. Pritchard, Wisconsin; L. L. Ratliff, Iowa; C. D. Roome, Canada; A. J. Roe, Illinois; J. W. Sparks, Illinois; A. G. Schloesser, Illinois; T. Stebbing, Wisconsin; J. O. Stanton, Illinois; E. LeRoy Turner, Iowa; H. J. Crumpton, California; J. W. Dawson, Iowa; R. R. De Witt, Illinois; A. C. Donovan, Missouri; A. A. Dye, Missouri; D. T. Douglass, Illinois; R. E. Egbert, Indiana; Wm. Eastman, Wisconsin; Wm. L. Everett, Illinois; George W. Frost, Iowa; John M. Furnas, Iowa; M. H. French, Iowa; T. D. Ford, Illinois; M. H. Garten, Illinois; C. H. Guibor, Colorado; H. A. Given, Illinois; E. T. Ingals, Illinois; Henry Jones, Illinois; J. E. Jones, Iowa; C. D. Knapp, Illinois; Thomas Kelley, Illinois; J. C. Lincoln, Iowa; T. P. Lark, Illinois; P. H. Leavitt Indiana; Robert La Grange, Iowa; Wm. T. Leonard, Wisconsin; R. J. Mitchell, Illinois; P. H. McElroy, Illinois; J. A. Masterston, Illinois; Robert McPherson, Illinois; George M. Macklin, Illinois; E. G. Minnick, Iowa; L. W. Thomas, Illinois; I. H. Taylor, Illinois; W. W. Williams, New York; C. A. White, Indiana; F. B. Wood, Michigan; E. B. Young, Iowa.

*Ad Eundem*—W. S. Baker, M.D., Illinois; Amos Knight, M.D., Michigan; M. H. L. Schooley, M.D., Missouri; D. L. Jewett, Illinois.

*Honorary*—Thomas M. Hess, Illinois; Zaccheus Bass, Vermont.

President J. V. Z. Blaney then delivered the charge to the graduating class:

*Gentlemen of the Graduating Class of* 1870–71:

You have this evening received the highest honors which this institution has the power to confer. Upon you has been conferred the Degree of "Doctor of Medicine" by the authority of the State of Illinois, and the Board of Trustees of Rush Medical College on recommendation of its Faculty, before whom you have passed a searching examination.

The conferring of this degree, and your acceptance of the diploma which certifies to these facts, invests you with several important privileges which I desire that you should fully appreciate, and at the same time with certain responsibilities which the Faculty of the College would wish fully to impress upon you before you bid your farewell to your Alma Mater.

These privileges are: 1st, that you are each of you endowed by all the influence, and with all the prestige which the College now holds in community, as proper persons to practice legitimate medicine; 2nd, that you are each of you admitted into the ranks of the Alumni of the Rush Medical College as members *de facto* and *de jure*—an association of which I feel warranted in asserting that, for the number of honorable and of accomplished medical practitioners which its rolls enumerate, cannot be excelled, if equaled by the Alumni Association of any institution of equal age in our country.

The responsibilities, voluntarily assumed by you, by your acceptance of these honors, and of your diploma, are numerous and obligatory. They are comprised in your obligations: 1st. To community, especially to that in which you may fix your residence; in this, that you will, at all times, be in readiness to respond to its urgent calls for your services, and be, as far as your circumstances and abilities permit, prepared in every way to respond to its needs. 2nd. To your profession, that you will use your best endeavors not only to keep pace with its advancement in all its departments, but also to make your own additions to its progress, and above all, that neither by word or deed you will swerve from the paths of legitimate medical science, or permit yourselves to be attracted by the allurements of the pocket, or of temporary success into the paths of empiricism, however bold their pretensions, or however promising their offers of preferment.

The response on the part of the class was made by Dr. C. A. White, as follows:

*Mr. President and Gentlemen of the Faculty:*

In behalf of the members of the twenty-eighth graduating class of this institution, I am before you to respond to the words which have just been spoken. To us the occasion is one of marked interest and great pleasure; nevertheless, when we come to look forward into the dim shadows of the future, and realize that we never shall again be permitted to assemble together, I imagine certain feelings of mingled pleasure and regret becoming developed within each of us at the idea of being now forever separated.

Here we have met in the capacity of teacher on the one hand, and of pupil on the other. We, of the latter, to accumulate and store up that knowledge which is to be our pleasure, our reliance, and our guide, while attempting to contribute to the welfare of humanity by the ministrations of the bounties of our chosen profession. From day to day, for the past eighteen weeks, we have been accustomed to assemble and re-assemble around the altar of science, within the majestic walls of this Æsculapian temple, to receive, from your laden lips, words of golden truth and meaning.

Here we have become thoroughly acquainted with the anatomical construction of our own bodies, the crowning work of the Creator's skill. We have seen the framework upon which no pains have been spared to make us perfectly familiar with its wonderful mechanism.

We have beheld, in wonder, the vital current coursing its way throughout the system, carrying with it life and health, and happiness to fair forms and hoary sages. We have been lost in rapt admiration, while gazing upon and contemplating those wondrous organs which constitute man's nervous system, the normal functional activity of which elevates him above the other works of the creation, gives to him supremacy over all the earth, and brings him into the nearest possible relation with the Creator himself.

We have passed the threshold of modern science, and have been admitted into its gorgeous labyrinth, deep down into the earth, midst its salts, its ores, and its precious metals; we have diligently searched each lofty mountain peak, and the broad and fertile vales between; we have been taken upon the eagle wings of science and wafted away through our own atmosphere to that of the sun, of the moon, and of the stars, and into ethereal space, all in one grand search for substances and forces which might be ascertained to possess specific influences over those conditions which we denominate disease.

But man, in his eagerness for knowledge, in his desire to unfold the works of the creation, has not stopped in these places content with those things only which he can realize by his natural endowments, but has developed for himself and brought to his aid numerous modes of investigation and observation, by which he is enabled, in many instances, to unlock the arcana of nature and behold her secrets and mysteries at will.

Thus, assisted by the power of the microscope, and by well-directed, unremitting labor, one of your own number, our worthy Professor Freer, has succeeded in discovering and demonstrating to the medical world an important element of the red blood corpuscle, which had escaped the acute observation of his scientific fathers and contemporaries, both in this country and in Europe. We have seen that, in keeping pace with the onward progress of the present age, the profession has been compelled to cast off much of the older paraphernalia of the healing art, and to substitute modes and measures more nearly in accordance with the teachings of modern science, rendering the profession once more nearly allied to an *actual* science, and less of an empirical art.

And thus, we recognize and welcome the great revolution which has taken place in the profession within the past quarter of a century—a revolution marked in character and of inestimable benefit; marked for the manifest changes which it has wrought by discarding entirely, or by restricting within proper limits, the use of those old-time therapeutic measures, the principal actions of which were those of extreme depletion only. Beneficial, by having replaced them in many instances by their very opposites, by administering the so-called medicines more sparingly, more judiciously, more scientifically; by economizing the powers of life, by relying more confidingly upon the vis medicatrix naturæ, to whose bidding alone we should respond, and to whose existence alone our efforts should be addressed, never presuming for a moment that human knowledge or skill can in the least improve its perfect works.

As students of nature, then, we go forth from this temple of learning, imbued with a thorough knowledge of the power of proper exercise, of rest, of pure air, of sunlight, of dietetic measures, and with the idea that, as yet, there are but very few, if, indeed any, specific remedies among medicines.

In view of the past, is it in the least vainglorious to suppose or even to predict that similar advances and discoveries, not to say absolute changes, from that which is now considered to be the truth, may take place in our time, and that some one or more of the members of the present class may develop and harness into the work of usefulness some of the yet hidden treasures of nature, thus each becoming a benefactor to the race for generations yet unborn?

The work is a great work. Its importance cannot be overestimated; and yet it is incomplete. We receive it from your hands, not as a finished something, but as something offering many opportunities for labor and observation before all of its secret avenues shall have been sufficiently traversed and all of its secret chambers fully explored. We have seen, 'tis true, that in many respects there is that which already approaches very nearly, or indeed, quite, to perfection; but there still remains sufficient fertile ground yet untilled, in which to spend our time and study most profitably.

"Labor with what zeal we will,
  Something yet remains undone,
Something uncompleted, still
  Waits the rising of the sun."

It is our intention, then, to enter the field laid out before us with a just appreciation of its magnitude; with a full determination to do honor and justice to the great cause in which we have become voluntarily enlisted, thereby proving to the world that the profession still exists, that it is self-sustaining, and that it will continue to be developed and grow until all the remotest nations of the earth shall realize and appreciate its beneficent influences in the highest degree of perfection, and join in the universal proclamation that its bounties and benefactions transcend all of man's other works.

At your hands, Gentlemen of the Faculty, we have received the great principles of medicine and surgery, constituting the grand framework of the temple in which our hopes and interests are completely enwrapped, and which it now becomes our duty to build, to ornament and adorn, each one for himself, in his own capacity, with the brilliant truths and triumphs of practical medicine and surgery. We acknowledge your competency to instruct; we acknowledge the ardor which you have each manifested in our behalf; we accept the numerous truths from your several branches, which, like so many beacon lights, are to guide and pilot us aright throughout our professional lives. We are aware of the responsibility which we owe to you, and we feel the weight of the responsibility which you have entrusted to us. For the lessons received from each of you, for your zeal as our teachers, for the energy which you have manifested in behalf of the entire class, for the confidence which you have bestowed upon us in the act of attaching your signatures to our diplomas, we tender our sincere thanks, and promise to be true to our *Alma Mater*, thus reflecting just and due credit and honor upon your worthy heads. We gladly receive and accept, and will never fail or forget to cherish, our diplomas from this institution; not merely for their own intrinsic worth or value as such, but as tokens from you and your honorable President, that, thus far, we have done well, and that henceforth we have your combined esteem and confidence, ever bidding us God-speed in the vast work spread out before us.

In receiving our diplomas we realize that we are elevated to an honorable position in the ranks of the alumni of this college, who, perhaps, constitute a majority of the working men of the profession throughout the Northwest, whose competency and skill have replaced happiness for grief in many households, and whose rectitude as men and physicians sustain, untarnished, the honor of your cherished institution. The feelings which are naturally generated between teacher and pupil are usually of such a nature as to constitute a firm bond of friendship throughout the lives of the parties. May this be the case with all of us.

And now, fellow-students, having pledged ourselves to be true to the institution which has given us professional birth and honors, let us be true to ourselves. In launching out upon life's waves to do our duty to ourselves and to our fellow-man, we are assuming the greatest of responsibilities. With clear heads and diligent feet we must pursue our course. And though it become obstructed with difficulties, we must not yield, must not turn to the one side nor to the other for an easier way, but with that courage which a consciousness of right and knowledge alone can inspire, surmount all difficulties. Let us go forth with sympathy in our hearts, with firm and unbiased minds, and with willing hands, deserving success and confidence, and we shall certainly command them.

Let us enter the arena of the profession with alone the armament of determination—the determination to work, to amass knowledge, to do justice, to conquer, to excel—for what should be our watch-word and our cry, if, indeed, it be not Excelsior?

Lastly, let us ever cherish and respect the lessons learned in this institution as our great landmark, so that we may look back with pleasure upon our school-days here, and proudly claim Rush Medical College as our *Alma Mater.*

Prof. Moses Gunn then delivered the Valedictory Address given below:

I was, lately, greatly interested in a copy of a rare old picture called "The School of Anatomy," intended to represent the first human dissection. The mingled sentiments which must have found place in the minds of the actors, in that then strange scene, were forcibly portrayed. Deathlike and expressionless lay the form which but recently had been the temple of the living spirit, and which, by that principle or force, had been actuated to deeds of might and valor, or had been the medium through which sentiments of love, hate and revenge had been manifested. Its deathlike aspect was heightened by the speaking attitudes and countenances of the awed, but intensely interested actors in that then unparalleled drama. The deep mysteries of the wonderful mechanism are about to be revealed. Not even the sacredness of the human form, the image of the great Creator, can longer withhold the overmastering desire for knowledge which has long yearned for the revelations of the analysis about to be made, until the barriers of human prejudice have at last fallen before it. But though mind triumphs over prejudice and directs the inquisitive scalpel in its invasion, still the idea that the ground on which they are entering is holy and not to be pressed with shodden feet, lends to the countenances of the actors a seriousness which adds to, and beautifies their earnestness. Sounding yet in their ears, "the temple of God are ye," they feel that they are entering the holy of holies, and with faces veiled with gravity and hands washed in innocency, they come up to their longed for, yet dreaded work. Strangely beautiful must have been the original scene which the artist has conceived and so successfully transferred to his canvas. Strangely it contrasts with our own rich and varied experience in anatomical research. Unfavorably does it contrast with the degree of gravity and earnestness with which we, at the present day, approach the subject. How little like a modern dissecting room scene! Mature and earnest men, who had already attained to the front rank in their profession, bent intently over that wonderfully laden table. What we now regard as the starting point in medicine, was to them an advanced study. Their starting point was not found in anatomical investigations, but in a strict empiricism. In fact, empiricism underlies the whole chronology of medical science. The effort to learn by experiment what was salutary, beneficial and curative in diseased and disordered states of the human system, was the first step in what became, in process of time, medical science. This system of empirical medicine, crude, uncertain and oftentimes erroneous as it was, and founded only in that innate disposition to preserve health, prolong life and increase the comfort of existence, led gradually and necessarily to the resultant desire to know the wherefore. The wherefore could only be found through a knowledge of the machine experimented upon, and that was enveloped in sacredness as well as shrouded in mystery. Resort, to study upon the structure and functions of that portion of the animal kingdom which most nearly resembled humanity, was naturally had; and thus the sciences of anatomy and its handmaid physiology, were commenced and attained some development, while ideas of pathology were yet as wild and visionary as the dream of delirium. But gradually and certainly grew the desire for, and love of knowledge. Like that divine sentiment which the immortal poet styles an appetite which grows by what it feeds upon, so the love of knowledge knows no satiety, but ever increasing in fervor and earnestness by additional acquisitions, like the mountain stream its course is ever onward with still increasing volume, till at last it shall be swallowed up in the vast ocean of truth. Thus the early dissections upon the lower order of animal being augmented knowledge and stimulated the desire therefor, till finally the sanctuary of the immortal soul, rendered still more mysterious and awful by the flight of that essence, was fearfully and tremblingly invaded. Solemnly, as if in the presence of the disembodied spirit, which might be viewing the desecration of its former tenement, were those dissections made. Nought, save an overpowering thirst for the especial knowledge revealed by those investigations, could have prompted and impelled them. And when that indifference which is the offspring of familiarity was attained, there still remained that prejudice to overcome which, even yet, is not fully conquered. But the science of human anatomy was established, and that of physiology commenced its vigorous growth, while close upon it followed a knowledge of pathological changes which soon began to take the form of a system. Then commenced comparative studies ranging from the lowest form of animal existence to the crowning work of earthly creations, for the purpose of developing to the highest degree a knowledge of that crowning work. Minute structure at last claimed attention, and the science of optics was invoked to lend its aid in the investigation. Histology of the present day is the result; and the anxious, earnest search after the yet unknown, through the object-glass of the one-fiftieth of an inch focus, with all the appliances and accessions of the modern microscope, only indicates our present status, not that to which we may yet possibly attain.

While this ever increasing scope and extension of study has gradually raised the standard of practical medicine and benefited mankind, it has been charged that its tendency has been towards a materialism which has often shocked spiritual sense and debased moral nature. Without here stopping to consider the truth of this statement, let us glance at some of the theories which have been advanced, and have seemed to warrant the charge, coming as they did from a scientific source, and claiming the merit of a refined philosophy. Not to go farther back than a period within my own recollection of scientific matters, the complacency of mankind in its manhood was not a little shocked by the statement that man had attained his present position at the head of animal existence, only as the last development in a progressive autogeny; that this self-development or plastic force in nature first brought forth the lowest forms of animal life; and that gradually acquiring power, and operating under ever improving circumstances and upon products which were ever advancing toward a higher degree of perfection, grade had succeeded to grade in an upward scale through the vast cycles of time, until mankind appeared upon the earth, the latest and most perfect in the series. That this last and crowning existence, in its individual embryonic life, passed rapidly through all the phases of a development representing successively all the inferior grades. That a constant improvement marked the ever succeeding generations, and that in the dim and distant future, out of the loins of the present type of humanity there might spring an order which should outrank it, as it now does the orang-outang and the gorilla.

Human pride had been well rebuked by the satires of Swift, but the satire was patent; here, however, under the garb of philosophy, was a theory which might well have been taken as a sarcasm more subtle and cutting than the trenchant wit of the eccentric Dean. But such was not its aim. It was put forth as a pure philosophy, and while its author protested against the charge of infidelity, and still spoke of the Creator and creative wisdom, he so shrouded the first great and Adorable Cause in such a mysticism as to be unrecognizable by the student of revelation, even though that revelation had been studied as an allegory with the light of science illuminating its pages.

Perhaps it may be considered by some a work of supererogation to reply to such vaporings, while others may consider a successful reply not within the range of human effort; nevertheless, it may not be amiss to look about us for some firm rock on which to place ourselves; some anchorage sure and reliable where our barque may ride in safety while the storms of a mad philosophy sweep ragingly past us.

Is the scale of animal being graduated with such regularity as to place man at the head, separated from the grade next below only by a single step or grade? Or, is there between man and the whole of the brute creation a chasm so deep and wide as to justify a faith in the statement that he was created but little lower than the angels? I propose to consider these two questions from stand-points which are more or less intimately related to your professional studies.

1st. From an anatomical stand-point. Is the step or grade from the highest type of the brute creation to the lowest type of the human family only equal to that which marks the difference between the highest, and the grade next below it of the brute creation? or, does the *tout ensemble*, as well as several specific anatomical points, mark a separation so wide as to break the series?

It seems as though there could be but one answer to the idea expressed in these two forms of the same question. There is no fear that the author of "The Vestiges of Creation" himself, would even for a single moment mistake, at the most casual glance, an average specimen of the lowest human type for the most perfect specimen of orang-outang or gorilla. That indescribable impression made by individual presence is so widely different in the two examples, that a willing proselyte to the so-called philosophic creed would stand back amazed at the difference which separates them. In the one he sees, emphasized in every attitude, glance, gesture and expression, only the brute; in the other, he cannot shut his eyes upon that spark of humanity which gives the hope of something beyond and better than the present, which lifts him to the pedestal on which stand, also, hope and aspiration.

But to pass from the whole to constituents; look at the form of the head, and its development, as compared with the face in the two specimens, and here, too, we see a longer step than any taken in the brute series. The length of the superior extremity; the four hands; and the facility, if not necessity, of quadrupedal locomotion in the brute, leaves a space between it and humanity too great to be spanned save by poetic fancy.

2d. From a psychological stand-point. Without denying to the brute creation a mental capacity superior to mere instinct, and even admitting that in some instances the brute may give evidence of a reasoning power that closely approaches true induction, still, in the results attained, he falls so far below the standard of the lowest humanity as to leave even a greater space between them, than that which separated them in anatomical development. The dominion over the "beasts of the field," which was given to man, according to revealed history, is enjoyed by the lowest example of the human race. His mental endowments enable him to construct implements and weapons for their capture, control and destruction. In this dominion, man, even in his lowest estate, proclaims his title to manhood, and indicates the vast space which separates him from the highest order of brutes. But in the ability to construct and speak a system of language; in the ability to learn and speak the language of other systems than his own; in his power to attain, by intercourse with, and instruction from others more learned than he, to still higher mental improvement, man asserts, incontrovertibly, his immense elevation above all other animal existences. No mere grade in a series is here marked; but a new series, with heavenward tendencies, is established. But it is neither in anatomical nor mental characteristics that the distance which separates man from all inferior orders is most clearly appreciated. Vast as it seems from the views which we have taken, let us proceed to our

3d. Stand-point of moral attributes. It is here that we shall obtain a still more convincing view of the intervening distance that, from our previous two stand-points, we have contemplated as separating humanity from all below it. It is here that we see, sharply drawn, the boundary lines between instinct and reason, because here, in obedience to his moral nature, man subordinates his instincts to his reason. The brute is a creature of instinct alone. Whatever of reason he may occasionally evince is in strict subordination to instinct. Natural instincts control and govern all his actions; and if he reasons at all, it is in furtherance of his instinctive desires. Man has, also, his instincts. Self-preservation, self-gratification, sexual love, hate, and revenge, are instinctive. But not as in the brute does man's reason minister to, and subserve these instincts. On the contrary, these natural instincts are subordinated to his reason; and just as he succeeds in such subordination, does he assert the divinity of his humanity. We sometimes say, figuratively, that a man is a brute; what do we mean by this speech, but that such a man is, to a great extent, following his natural instincts—failing to control them by, or subordinate them to, his reason? There is, probably, no example of humanity however low (unless bereft of reason) which does not, in a greater or less degree, control and govern his instincts. This is the prerogative of humanity. The power to subordinate instinct to reason is absolutely wanting in the brute; in fact, as has been already stated, whatever of reason the brute may have, is the slave to animal instinct. Instinct is the master in the brute, but the subject in man. And man, in his mastery over his instincts, where they interfere with his higher aspirations, asserts his humanity and vindicates his claim to immortality. Whatever belief may be entertained as to my former two positions, whether coinciding with the sentiments which I have advanced, or regretfully distrusting them, it would seem as if there could be no doubt upon this last proposition. It is here that we *feel* ourselves to be more than the beasts which perish. In our love of the beautiful and the true, in our admiration of the mighty and the noble, in our appreciation of the grand and the sublime, in our conception of the vast and the awful, in our desire to grasp the infinite, in our adoration of the Omnipotent, in our aspirations to a still higher life, we realize our God-given dignity and lay hold upon our heavenly birthright.

From this expression of faith which rises in my own mind from a contemplation of the facts to which I have called your attention, let us recede to a strictly logical conclusion, and ask what they actually prove? To this I reply: They prove that the order of animate being is not an unbroken series of uniform gradation; and, consequently, it affords no evidence of an autogenous force, the assumption of which is based upon such gradation.

This doctrine of an ever operative plastic force, which the author of the Vestiges of Creation believed had first brought forth the lowest forms of animal life, and improving upon its own work by a sort of cumulative energy, and the operation of elective affinities through the long ages of the earth's history until the present grade was reached, of course, regards that force as merely an inherent property of certain forms of matter. It is consonant, if not identical, with the theory of Darwin as to the origin of species; and an earnest advocate of Darwinism, Prof. Huxley, delivered, something over two years since, a lecture entitled "The Physical Basis of Life," which, as is indicated by its title, recognizes the same inherent spontaneous principle, property or force. I mention this effort of Prof. Huxley, not for its singularity—for he is but one of a numerous class of philosophers who entertain, and strenuously advocate, both in season and out of season, similar views—but because it has attracted more attention, both in this country and in Europe, than any other similar paper published for a long time.

Physical basis of life is used as entirely synonymous with protoplasm; and the meaning of the author would not be distorted by the expression: protoplasm is the physical basis of life. Now, if by protoplasm was merely meant a certain kind of material substance or pabulum, which constituted a physical basis or substance necessary to the support of life, a very correct idea would be very capitally formulated. And indeed, the way in which he talks some of the time in this lecture is, certainly, entirely consonant with such a meaning of the term; for example, when he says that "plants can manufacture fresh protoplasm out of mineral compounds, whereas animals are obliged to procure it ready-made, and hence, in the long run, depend upon plants. An animal cannot make protoplasm, but must take it ready-made from some other animal, or some plant—the animal's highest feat of constructive chemistry being to convert dead protoplasm into that living matter of life which is appropriate to itself." He also says: "this present lecture, whatever its intellectual worth to you, has a certain physical value to me, which is conceivably expressible by the number of grains of protoplasm and other bodily substance wasted in maintaining my vital processes during its delivery."

He contemplates, also, the recourse which he shall have to savory roast mutton, to supply this waste of protoplasm, whereby ovine protoplasm shall become human protoplasm. His fancy, too, dwells upon the pleasure he might derive by supping upon lobster, but for the fear of putting his digestive organs to too severe a test, and thus converting crustacean protoplasm into the human article; and further, his possible shipwreck, and the feeding of lobster upon himself, whereby the compliment would be returned, and Huxley protoplasm become transubstantiated into crustacean protoplasm.

Now, all this might be good orthodox common sense and philosophic physiology. But Prof. Huxley takes good care that we shall have no excuse for so construing it; for he compares the properties of protoplasm and the changes which it may undergo to the changes wrought by the chemist when he effects that combination of oxygen and hydrogen which results in the production of water, and asks, "what better philosophical status has 'vitality' than 'aquosity'?" And again, he says, "If the properties of water may be properly said to result from the nature and disposition of its component molecules, I can find no intelligible ground for refusing to say that the properties of protoplasm result from the nature and disposition of its molecules. But I bid you beware that," he frankly admits, "in accepting these conclusions, you are placing your feet on the first rung of a ladder which, in most people's estimation, is the reverse of Jacob's, and leads to the antipodes of heaven. It may seem a small thing to admit that the dull vital actions of a fungus or a foraminifer are the properties of their protoplasm, and are the direct results of the nature of the matter of which they are composed. But if, as I have endeavored to prove to you, their protoplasm is essentially identical with, and most readily converted into, that of any other animal, I can discern no logical halting place between the admission that such is the case, and the further concession that all vital action may, with equal propriety, be said to be the result of the molecular forces of the protoplasm which displays it. And if so, it must be true, in the same sense and to the same extent, that the thoughts to which I am now giving utterance, and your thoughts regarding them, are the expression of molecular changes in that matter of life which is the source of our other vital phenomena."

I cannot refrain from the remark right here, though, of course, it is not offered as an argument, that, judging by the quality, Prof. Huxley's thoughts on this last named point might very possibly, figuratively speaking, emanate solely from protoplasmic molecular changes on a level with the "dull vital action of a fungus or a foraminifer," though it is to be hoped that the thoughts of his audience in reference thereto may have had a higher origin; and that molecular change was the instrumental process through which they took, not the cause of their taking, form. I have made a quotation to show you, not only the nature of the mental protoplasm which Prof. Huxley offers, but also, that he does not disguise it by either flavor or condiment. He, evidently, administers his pills without sugar-coating. For this boldness let us sincerely render him our thanks.

The plain unvarnished question which is here forced upon our consideration is as follows: is protoplasm, in virtue of its physical and chemical composition, possessed of an independent organizing force? If so, it may well claim the prerogatives which have heretofore been awarded to vitality. If so, a mass of protoplasm ought to rise by its own inherent and independent energy or force into organizing activity, and assume, unaided, organic form, and display all the phenomena of life. Does it ever do so? Prof. Huxley and the school of philosophy of which he is a bright and shining light, have never shown us a single example of such action, and until they can do so, their protoplasm lacks its physical basis, and their claim is not worth a moment's credence, or hardly a moment's consideration.

The simple facts, that are at present established, are, that the so called protoplasm is a material which living organisms of the vegetable kingdom make by selecting and compounding elements from mineral compounds. This process of making protoplasm by living vegetable organisms, is a function possessed only by them. Art has never duplicated the process. There is no known instance of the elements combining to form protoplasm by their own power or elective affinity; but in every known instance a previously existing living organism seizes upon the elements and combines them to form protoplasm. Protoplasm when thus formed has no power or force by which, unaided, it can rise to a higher dignity; but it may be acted upon by a living organism, or it may fall under the influence of chemical force. If acted upon by the former, it becomes organized and forms a constituent of the organism; if not so acted upon, *i. e.*, if plant protoplasm, separated from the organic plant, or Prof. Huxley's roast mutton protoplasm is not consumed by some animal and thus brought in contact with a living organism, it becomes a prey to chemical force, and by it is resolved into its original elements, which elements may be selected by some plant and reconstructed into plant or vegetable protoplasm. Before protoplasm or protoplasmic elements can take the first step upwards in development, it or they must be selected and appropriated by a living organism; and in every succeeding upward step until it reaches the grade of living organic tissue, protoplasm derives whatever of force it displays from the organism under whose auspices it is being advanced and into which it is becoming incorporated. However perfectly elaborated protoplasm may be, if left to itself, unaided by the influence of a living organism, it loses whatever of energy or force it may thus far have obtained, and, under the action of chemical laws, begins a downward course. The exceptions to this rule will be found in the ovum of an animal and in the seed or germ of a vegetable. In these instances the egg or seed protoplasm has enjoyed an additional advantage from impregnation, which gives it a power of remaining for a variable period of time in *statu quo* until, under favorable circumstances, it springs into independent, organizing action. If such favorable circumstances should not surround it, chemical force at last overpowers the latent independent spark, and, by its action, sends it down again to the bottom of the series

But in this endowment of the ovum or seed with a certain degree of independent force, another interesting and important process is seen, which sheds a flood of light upon our subject. For example, the egg of an oviparous animal is a very capital specimen of animal protoplasm. It may be consumed and appropriated by another animal. Prof. Huxley, for instance, may partake of it in lieu of roast mutton protoplasm, and in the changes which it will undergo, and in the support to his exhausted energies which it will afford, it will fully vindicate its title to first-class protoplasm, even though it may never have enjoyed the advantage of impregnation. But what would be its fate, if, under the last supposition, an effort were made to hatch a chick from it? The answer is found in two simple words, viz.—*addled egg*. Where, then, are those properties of this protoplasm which result from the "nature and disposition of its molecules," which Prof. Huxley could find no intelligible ground for discrediting, and which to him stand in place of all vital action? Has not the first rung in his anti-Jacob's ladder broken under the step? and is it not fortunate that it has broken? for he acknowledges that, in most people's estimation, it reaches not to heaven, but to the other place, as Hamlet has it.

But let us suppose that another specimen of egg protoplasm has been impregnated and placed under favorable circumstances for hatching. Mark the interesting and mysterious change! A new being springs into existence, endowed with all the powers of the parent. Whence the force which this mass of protoplasm now displays and which it did not possess before impregnation? Some animals deposit their unimpregnated eggs in places favorable to their development; now if these eggs are not found and impregnated by the male, they soon decay under the operation of chemical law, although they are surrounded by the physical forces which are not only favorable to, but necessary for their development; if, however, the male finds them and performs his function, they thereby become the recipients of a force through which they spring into energetic, organizing activity. Whence, and what, again I ask, is the force which this mass of protoplasm now displays? It must be acknowledged that, as mere protoplasm, it had it not; nor can it be claimed that the impregnating fluid is protoplasm; and if it were protoplasm, there being only contact between it and the egg protoplasm, and no mingling of the two forms of protoplasm, the vivifying influence which is imparted by the one and received by the other must be regarded as a force which the most perfect form of protoplasm did not, by the "nature and disposition of its molecules," possess. But as impregnating fluid is not protoplasm, the vivifying force must be looked for elsewhere than in protoplasm, notwithstanding the "nature and disposition of the molecules" of that substance. Protoplasm is a good physical basis or material for the support of life; but before it can live and grow it must become vivified from another source than itself.

Another disciple of the so-called new philosophy is Prof. Barker, of Yale College, who delivered a lecture in New York before the American Institute early in 1869, which elicited from the press of the country pretty general discussion. The subject of this lecture was: *The Correlation of Vital and Physical Forces.* Surely this is a pretentious title. To establish a reciprocal and convertible relation between physical forces and that mysterious force which constitutes life, and for which the term vital is so apt an adjective that even the new philosophers themselves continue to use it while denying the quality which it represents, is, certainly, a work which challenges boldness of enterprise and confidence of power. But Prof. Barker's boldness and enterprise are first displayed in an attempt to level vitality down to the grade or status of a physical force. He says: "Every particle of matter within the body obeys implicitly the laws of chemical and physical attractions. No overpowering or supernatural agency comes in to complicate their action, which is modified only by the action of the others. Vitality, therefore, is the sum of the energies of a living body both potential and actual."

Now, is it true that the matter of a living body obeys implicitly the laws of chemical and physical attractions? If so, what law of attraction does the matter of a dead body obey? Certainly the matter acts very differently in the two conditions. It is not unrestrained chemical and physical attractions in both instances; and no one can deny that a dead organism is the seat of chemical and physical actions alone. And, if this is true, there must be an overpowering force in the living body which does exert a restraining and controlling inflence over chemical and physical attractions.

The manner and mode of death, and the circumstances which surround the change from life to death in some rare instances, afford a striking illustration of this fact. For instance, a soldier upon the field of battle, in full health, kneeling upon the right knee with the left foot advanced and firmly planted, while taking aim in this position receives a shot which instantly destroys life, and leaves the body rigid in the position described. Here is the same physical body or substance, vigorously living in one instant, in the next dead. Now let us contrast the changes which begin to occur immediately after death, with those which were in active operation just before. And first let us notice a change which is due to the physical force, gravitation. The fluids of the body begin, in obedience to this force, to settle into the lowest portions; and so far as anatomical structure will permit, they will find their way thither. This hypostatic accumulation of fluids is familiar to all who have had much to do with the cadaver. But while life was present no such gravitation of fluids took place. On the contrary, the blood in the veins below the heart and the lymph in the lymphatics and thoracic duct mounted upwards in direct opposition to the force of gravity.

In reference to this point, Dr. Beal asks: "Does the tree grow away from the earth, or its roots into it, in obedience to the laws of gravitation? * * * * Of course, it will be said that capillary attraction, osmose, and other forces, contribute in a highly complex manner to bring about these results; but every one at all acquainted with the subject knows, that the facts have not been, and cannot be, explained." Dr. Beal might have added that the dead organism of a tree afforded the same physical facilities for capillary attraction and osmose, as did the living; yet, here, these physical forces fail to operate; no sap laden with the elements of plant protoplasm mounts toward the topmost branches.

As to the operation of chemical force, our poor soldier who fell at Seven Pines, under the favoring influence of a Virginia summer, became at once the seat of a vigorous breaking up of former combinations, and a recombination of the liberated elements. But before death these combinations were undisturbed. Elements of tissues and fluids which began to shun one another immediately after death, before that change, had remained in a firm and harmonious combination. Some force must have been present and operative, before death, which *was* "overpowering," and which *did* "complicate" the action of "chemical and physical attractions."

Prof. Barker's "therefore," then, falls to the ground, and vitality is something more than the sum of physical energies. Vitality is not the sum of any energies whatever, but the mainspring and source of those energies. It is the cause of energy, not its manifestation.

Prof. Barker illustrates, or rather adduces instances of, the correlation of physical forces, i. e., their reciprocal relation to one another, as evinced in the conversion of heat into motion and motion into heat; heat into electricity and electricity into heat, etc. He then shows that the various changes going on in a living animal organism under the inspiration of, or in obedience to, vital force, are accompanied by, or result in, the development, simultaneously, of certain physical forces; as for example, heat, motion, and in some instances, electricity. He shows, also, that the brain changes which take place in mental operations, develop heat; and even goes so far as to measure the different degrees of heat developed by different mental operations or conditions. And this he calls a correlation of physical and vital forces! Surely this is correlation with a vengeance! Such a correlation is a correlation which correlates in one direction only. It sounds very much like the proposed division of game between the pale faced and Indian hunters, who together had shot only a turkey and a crow. Pale face says: "I'll take the turkey and you may take the crow; or, you may take the crow and I'll take the turkey." But the copper skin was smarter than Prof. Barker deemed his audience, for he replied: "It's turkey you and crow me all the time." With Prof. Barker, it is vital force developing physical force all the time, with no instance of physical force begetting vital force.

Until the disciples of the new philosophy are able to convert light, heat, electricity, motion, gravitation, and other forms of physical and chemical attraction, one or more, or all of them, into vital force, there can be no proof of a correlation between physical and vital forces. As yet, they have not been able to accomplish this; and we must, for the present at least, continue to procure our new supplies of living organisms by the same old method; each must beget its kind according to methods which have obtained from the beginning. We must wait patiently till the new philosophy succeeds in taking its first sure step in this direction. That accomplished, we can, perhaps, look confidently forward to the time when additional skill and increased facilities will enable them to reproduce extinct forms. Then museums of fossil remains will indeed become *passeé;* for the trilobite, the plesiosaurus, the megatherium, and the mastodon, reappearing under the magic touch of science in all their pristine vigor and beauty, shall lie down together, and a little philosopher shall lead them! Then, forsooth, the millennium of science will have dawned upon a startled world.

Gentlemen Doctors: It may, perhaps, strike you that this lecture is but a dry morsel to offer you on this parting occasion; that the last hour of the session should not have been devoted to the consideration of a scientific subject, but rather have been used for the purpose of giving such sage advice upon general subjects as my experience might warrant. How, for instance, to so deport yourselves as most surely to insure professional success. I confess that I should shrink from grappling with this subject. Professional success will depend entirely upon your individual selves; upon your individual adaptiveness to the discharge of professional duty; and according to this adaptiveness, will the degree of success to which you may attain, be measured.

The parting injunction, which, above all others, I would lay upon you, is expressed in these four words: *endeavor to deserve success.* Your endeavors in this direction must relate to the earnest and thoughtful cultivation of science; to the diligent and honest discharge of your duties to your patients; to the equally honest, and, sometimes, fearless performance of your duty to community.

Thus far, it is probable, that you are all debtors to the world, having received from, more than you have been enabled to render to, your kind. I know of no one thing more healthy in its influence upon individual character than a full appreciation of this fact, if it stimulate to efforts to transfer the balance to the credit side of your account.

And, gentlemen, when finally you shall

> "Wrap the drapery of your couch about you
> And lie down"—

in your last, long sleep—that your account shall show that you have lived for the betterment of the world, is the earnest hope of your Alma Mater.

After the valedictory, Dr. A. L. Buchan, of Wisconsin, stepped forward, and, in a very impressive speech, presented an elegantly carved armed chair to Professor Rea, lecturer on Anatomy.

The chair is made of elegantly finished black walnut, of beautiful, easy-chair design, with carved skulls upon the arms. Professor Rea, at the close of the presentation speech, responded in the following manner, his remarks being received with applause both loud and long:

There left this institution at the end of the session of 1866–7 a class that I have always regarded as the best we have ever graduated. But now, I think, all things considered, looking at everything, I think that will have to step aside, and the class of 1870–71 take its place. For you will be pleased to learn, that we reckon students here as they are anatomists, the time having passed when anything else was counted. Here

> Anatomy makes the man, the want of it the fellow,
> The rest is but leather and prunella, and dog leather at that, with a slight mixture of ipecac, oxytoccia, waxed ligatures and "what's the matter." This beautiful testimonial, gentlemen, takes me quite by surprise, so much so that I fear I shall forget the speech I had prepared for the occasion. You have noticed, perhaps, that *my* speechmaking depends very much on my *subject*. When I first saw this splendid thing approach, you will excuse me if I felt a little "chairy," for it seemed to me there was some "skullduggery" about it.

In the selection of this beautiful, durable and valuable memento, gentlemen, you have aptly embodied the suggestion that the many years I have stood for anatomy in this institution, is rapidly bringing me to that point of time in which your elegant gift will become more and more necessary to my comfort. Be assured, I prize it more than any present I ever received, and shall keep it among my choicest treasures, not alone on account of its intrinsic value, but of the peculiarly pleasant circumstances under which it is received; and when the gray hairs and tottering steps shall call more loudly for longer recesses from labor, pillowed in these soft cushions, embraced by these kindly representatives of your strong and generous arms, my hands resting on these artistic emblems of our past associations, one of the happiest thoughts of these hours of sweet rest will be that it came from the magnanimous hands of the class of 1870–71.

Gentlemen, I thank you.

President Blaney then announced that he had resigned his position as head of the faculty, and that at the recommendation of the faculty, the trustees had appointed Prof. Joseph C. Freer to fill the vacant position. The new President delivered a short address on being inducted into his chair.

A prize of $25 was bestowed on Dr. W. T. Montgomery, of the graduating class, for the best anatomical preparation.

This premium was offered by Chas. T. Parkes, M.D., the Demonstrator of Anatomy. It is but justice to the several competitors to say that the Committee of Award paid a high tribute of commendation to all the specimens of the work brought up for inspection.

The benediction was pronounced by the Chaplain, and the "Commencement" of 1870–71 was *fait accompli*.

